# Clinical classification of cardiovascular tumors and tumor-like lesions, and its incidences

**DOI:** 10.1007/s11748-013-0214-8

**Published:** 2013-03-05

**Authors:** Jun Amano, Jun Nakayama, Yasuo Yoshimura, Uichi Ikeda

**Affiliations:** 1 Department of Cardiovascular Surgery, Shinshu University School of Medicine, 3-1-1 Asahi, Matsumoto, Nagano Japan; 2 Department of Molecular Pathology, Shinshu University Graduate School of Medicine, 3-1-1 Asahi, Matsumoto, Nagano Japan; 3 Department of Orthopaedic Surgery, Shinshu University School of Medicine, 3-1-1 Asahi, Matsumoto, Nagano Japan; 4 Department of Cardiovascular Medicine, Shinshu University School of Medicine, 3-1-1 Asahi, Matsumoto, Nagano Japan

**Keywords:** Cardiac tumor, Metastatic cardiac tumors, Classification, Incidence, Cardiac myxoma

## Abstract

Tumors of the heart and the great vessels are very rare disease, and there are many disorders such as tumors originated from the heart and great vessels, metastatic tumors, and tumor-like lesions which do not fit into the usual concept of tumor or neoplasm; thus, it is very difficult to classify these tumors. We proposed a new classification of cardiovascular tumors for clinical use based on the accumulated biological analyses and clinical data of the reported literatures and our own study as benign tumors, malignant tumors, ectopic hyperplasia/ectopic tumors/others, and tumors of great vessels, with reference to the series of Atlas of tumor pathology of the Armed Forces Institute of Pathology and the recent World Health Organization classification of cardiac tumors issued in 2004. More than 50 disorders have been reported as tumors originated from the cardiovascular system, and various metastatic tumors from nearby organs, distant lesions, and intravascular extension tumors to the heart were reported. Based on the new classification, we reviewed epidemiology and incidence of cardiovascular tumors. Metastatic tumors are more frequent than tumors originated from the heart and great vessels, and cardiac myxoma is the most frequent tumors in all cardiac tumors.

## Introduction

Tumors of the cardiovascular system, benign or malignant, are very rare disease, and there are many disorders which do not fit into the concept of tumor or neoplasm; thus, it is very difficult to classify the tumors of the heart and great vessels. As tumors of other organ, tumor of the heart and great vessels are classified as tumors originated from the heart and great vessels, or secondary/metastatic tumor with invasion nearby organs such as lung or metastatic tumors that occur in the distant organ. In addition, tumors originated from the heart are classified by the site of tumor location such as tumor of the heart, cardiac septum, pericardium, or great vessels, and classified by cell type constituting the tumor such as hyperplasia, hamartoma, cyst, or benign or malignant, and also classified by histological features such as mesenchymal, epithelial, and serous membrane (mesothelium). However, there is no established standard method of classification of tumors of the heart and great vessels up to now. In this review, we propose a new clinical classification for tumors of the cardiovascular system, and summarize its epidemiology and incidence from the reported literatures and our own study.

## Classification of tumors of the heart and great vessels

Tumors originated from the heart and great vessels are found at an incidence of about 0.02 % of autopsy. Among these cardiovascular tumors, 70 % of these tumors and most of the surgically excised tumors are benign [1]. Since there is no counterpart tumor in other organs or lack of histogenetical and pathological evidence of true tumor for papillary fibroelastoma, histiocytoid cardiomyopathy, lipomatous hypertrophy of the interatrial septum or cystic tumor of atrioventricular node, and others, pathological classification is very difficult for these tumors [2]. And also, it seems difficult to classify the tumors by histological type, because there are many tumors whose origin is not clear like tumors appear in other organs and tissues. A typical example is that in cardiac myxoma, origin of cardiac myxoma and tumor characteristics is not yet elucidated, and it contains various cell components like epithelium, endothelium, nerve, and undifferentiated mesenchyma which potentially differentiate into many tissues such as blood vessels, glandular structures, bones, and foci of extramedullary hematopoiesis [3]. Also, sarcoma is a malignant cardiovascular tumor, and despite very low incidence, many types of sarcomas which found in other organs were reported in the literatures [4]. However, there is so-called undifferentiated sarcoma exists that cannot determine a certain tendency to differentiate into cells or tissues even if using the latest diagnostic technology such as immunohistochemistry, electron microscopy and genetic analysis [5].

Because there are many cardiac tumors whose origin is not yet determined and cell differentiation was not elucidated, it is very difficult to systematically classify these tumors. According to the well-known classification of “tumors of the heart and great vessels” (Armed Forces Institute of Pathology: AFIP) published in 1996 [3] (Table 1), cardiac tumors including both cardiac tumors and pericardial tumors are classified into benign or malignant cardiac tumors. Sarcomas of the aorta and pulmonary artery, sarcomas of the inferior vena cava, and leiomyomatosis of veins are classified in different categories. Benign cardiac tumors are further classified as tumors of unknown histogenesis, tumors of cardiac muscle, tumor of fibrous tissue, vascular tumors and tumor-like lesions, tumors and proliferations of fat, tumors and tumor-like lesions of mesothelial cells, tumors of neural tissue, tumors of smooth muscle, heterotopias, and tumors of ectopic tissue. And malignant cardiac tumors are classified as sarcomas, malignant germ cell tumors, hematologic tumors, granulocytic sarcoma, mesothelial malignancies, and metastatic tumors to the heart.

**Table 1:** Classification of “tumors of the heart and great vessels” by the Armed Forces Institute of Pathology

Benign cardiac tumors
Tumors of unknown histogenesis
Myxoma
Papillary fibroelastoma
Tumors of cardiac muscle
Rhabdomyoma
Histiocytoid cardiomyopathy (purkinje cell hamartoma)
Miscellaneous hamartomas
Tumors of fibrous tissue
Fibroma
Solitary fibrous tumor of pericardium
Benign fibrous histiocytoma
Inflammatory pseudotumor
Vascular tumors and tumor-like lesions
Varix
Hemangioma
Hemangioendothelioma
Hemangiopericytoma
Lymphangioma
Tumors and proliferations of fat
Lipomatous hypertrophy, interatrial septum
Lipomatous hamartomas of cardiac valves
Lipoma
The fatty heart
Tumors and tumor-like lesions of mesothelial cells
Mesothelial cysts
Mesothelial/monocytic incidental cardiac excrescences
Mesothelial papilloma
Tumors of neural tissue
Granular cell tumor
Schwannoma/neurofibroma
Paraganglioma
Tumors of smooth muscle
Leiomyoma
Intravascular leiomyomatosis
Heterotopias and tumors of ectopic tissue
Bronchogenic/foregut cysts
Tumors of the atrioventricular nodal region
Teratoma
Ectopic thyroid
Intrapericardial thymoma
Malignant cardiac tumors
Sarcomas
Angiosarcoma
Malignant fibrous histiocytoma
Unclassified sarcoma
Myxosarcoma
Fibrosarcoma
Leiomyosarcoma
Rhabdomyosarcoma
Osteosarcoma
Synovial sarcoma
Malignant schwannoma (malignant peripheral nerve sheath tumor)
Malignant mesenchymoma
Malignant hemangiopericytoma
Kaposi’s sarcoma
Malignant germ cell tumors
Hematologic tumors
Lymphoma
Granulocytic sarcoma
Mesothelial malignancies
Malignant mesothelioma
Metastatic tumors to the heart
Sarcomas of the aorta and pulmonary artery
Luminal (intimal) sarcoma
Unclassified sarcomas
Malignant fibrous histiocytoma
Angiosarcoma
Osteosarcoma
Chondrosarcoma
Leiomyosarcoma
Malignant mesenchymoma
Mural sarcomas
Leiomyosarcoma
Angiosarcoma
Malignant fibrous histiocytoma (MFH)
Unclassified sarcomas
Sarcomas of the inferior vena cava
Mural leiomyosarcoma
Luminal (intimal) sarcoma
Leiomyomas of veins

Modified from reference [3]

The recent classification by WHO in 2004, tumor of the heart are divided into three categories: benign tumors and tumor-like lesions, malignant tumors, and pericardial tumors [4] (Table 2). In benign tumors, tumor were classified as tumor showing differentiation into muscle cells such as rhabdomyoma, adult cellular rhabdomyoma, hamartoma of mature cardiac myocytes, and histiocytoid cardiomyopathy. Cardiac myxoma and papillary fibroelastoma are classified as pluripotent mesenchymal origin, and cardiac fibroma and inflammatory myofibroblastic tumor were classified as tumor showing differentiation into myofibroblastic cell. Other benign tumors are vascular tissue origin as hemangioma, fat tissue origin as lipoma, and congenital cystic lesions in the atrioventricular node as cystic tumor of atrioventricular node. This classification includes relatively high incidence of benign cardiac tumors, however, tumors of low incidence such as tumors of neural cell differentiation or smooth muscle cell differentiation are not included. Most prominent differences of WHO classification from AFIP classification are classification of malignant tumors. Firstly, epithelioid hemangioendothelioma, formerly classified as benign tumor has been classified as malignant tumor, and secondarily undifferentiated sarcoma, which has been classified as tumor of unknown origin, is united to form one disease as malignant pleomorphic fibrous histiocytoma (MFH)/undifferentiated pleomorphic sarcoma subtype. Other features of WHO classification are malignant mesenchymoma, osteosarcoma, chondrosarcoma, and many other sarcomas were not included as an independent sarcoma, but included in MFH/undifferentiated pleomorphic sarcoma, and tumor that had been referred to as myxosarcoma specific for heart was classified as a subtype of myxoid fibrosarcoma.

**Table 2:** WHO histological classification of tumors of the heart

Benign tumors and tumor-like lesions
Tumors of muscle cell differentiation
1. Rhabdomyoma
2. Histiocytoid cardiomyopathy/purkinje cell hamartoma
3. Hamartoma of mature cardiac myocytes
4. Adult cellular rhabdomyoma
Pluripotent mesenchymal tumor
1. Cardiac myxoma
2. Papillary fibroelastoma
Haemangioma
Tumors myofibroblastic cell differentiation
1. Cardiac fibroma
2. Inflammatory myofibroblastic tumor/Inflammatory pseudotumor
Cardiac lipoma
Cystic tumor of atrioventricular node
Malignant tumors
Cardiac sarcomas
1. Angiosarcoma
2. Epithelioid hemangioendothelioma
3. Malignant pleomorphic fibrous histiocytoma (MFH)/undifferentiated pleomorphic sarcoma
4. Fibrosarcoma and Myxoid fibrosarcoma
5. Rhabdomyosarcoma
6. Leiomyosarcoma
7. Synovial sarcoma
8. Liposarcoma
Cardiac lymphoma
Metastatic tumors to the heart
Pericardial tumors
1. Solitary fibrous tumor
2. Malignant mesothelioma
3. Germ cell tumors
4. Metastatic pericardial tumors

Modified from reference [4]

*WHO* World Health Organization

Since the AFIP classification of the heart, pericardium, and great vessels has been widely used, we propose a new classification based on AFIP classification, with reference to the WHO classification and taking into account the recent findings of cell differentiations and clinical importance [6] (Table 3). Cardiac tumors are classified as benign tumors, malignant tumors, and ectopic cardiac tumors and other tumors. Benign tumors are classified into cardiac tumors and pericardial tumors. Malignant tumors are classified as tumors originated from the heart, metastatic cardiac tumors, intravascular tumors with extension to the heart, and malignant pericardial tumors. The tumors of ectopic cardiac tumors and other tumors are tumors of unknown origin, ectopic other tissue formation within the heart, tumor-like lesions due to immunological or hematological disorders, and apparently no tumor lesion but should be differential diagnosed. IgG4-related sclerosing disease are included in this classification as a cardiac tumor, since this disorder are reported to be systemic immunological disorder and forming lesions in the salivary glands as well as pancreas and tumor-like lesion in the heart. In addition, the immunodeficiency-associated lymphoproliferative disorder due to the recent spread of AIDS has been included in this category. Thrombus is not a true tumor, and it was not included in the AFIP classification. We picked up thrombus as “other disease”, because it is useful for routine clinical differential diagnosis when intracavitary or intravascular lesions is encountered.

**Table 3:** Classification of tumors of the heart and great vessels: new classification based on the cell differentiation and clinical importance

Benign tumors
I. Cardiac tumors
Cardio-specific tumors
1. Cardiac myxoma
2. Papillary fibroelastoma
Tumors and tumor-like lesions of muscle cell differentiation
3. Rhabdomyoma
4. Leiomyoma
5. Histiocytoid cardiomyopathy/purkinje cell hamartoma
Tumors of fibroblast and myofibroblast cell differentiation
6. Cardiac fibroma
7. Inflammatory myofibroblastic tumor/inflammatory pseudotumor
Tumors of vascular vessel and lymphatic vessel differentiation
8. Hemangioma
9. Angiomyolipoma
10. Hemangiopericytoma
11. Lymphangioma
Tumors and tumor-like lesions of adipocyte differentiation
12. Lipomatous hypertrophy
13. Lipoma
14. Lipomatous hamartoma of cardiac valves
Tumors of nerve cell and nerve sheath differentiation
15. Granular cell tumor
16. Paraganglioma
17. Neurofibroma
18. Neurinoma/Schwannoma
Other cardiac tumors and tumor-like lesions
19. Teratoma
20. Hamartoma
21. Cystic tumor of atrioventricular node
II. Pericardial tumors and tumor-like pericardial lesions
22. Solitary fibrous tumor of the pericardium
23. Mesothelial papilloma
24. Intrapericardial thymoma/pericardial thymoma
25. Pericardial cyst
Malignant tumors
I. Cardiac tumors originated from the heart
1. Angiosarcoma
2. Cardiac intimal sarcoma
3. Epithelioid hemangioendothelioma
4. Malignant pleomorphic fibrous histiocytoma (MFH)/undifferentiated pleomorphic sarcoma
5. Osteosarcoma
6. Rhabdomyosarcoma
7. Leiomyosarcoma
8. Fibrosarcoma/myxosarcoma
9. Synovial sarcoma
10. Liposarcoma
11. Malignant schwannoma
12. Malignant lymphoma
II. Metastatic cardiac tumors
1. Direct invasion
2. Metastasis
III. Intravascular extension tumors
1. Renal cell carcinoma
2. Leiomyomatosis of veins
IV. Pericardial tumors
1. Malignant mesothelioma
2. Metastatic tumors
3. Leukemic infiltration of the pericardium
Ectopic hyperplasia/Ectopic tumors · Others
1. Aberrant goiter/Thyroid heterotopia/ectopic thyroid
2. Bronchogenic cyst
3. Mesothelial/Monocyte incidental cardiac excrescence (MICE)
4. Blood cyst
5. IgG4-related sclerosing disease
6. Immunodeficiency-associated lymphoproliferative disorder
7. Wegener’s granulomatosis
8. Calcified amorphous tumor
9. Thrombus
Tumors of the great vessels
1. Aortic tumors
2. Pulmonary artery tumors
3. Tumors of the great veins

Modified from reference [6]

Tumors of the great vessels are further rare tumors compared to the cardiac tumors. Most of them have been malignancies in the aorta, pulmonary artery, and vena cava, and the high incidence in the venous system. The classification of the tumors of great vessels is not well established because of its very low incidence and lack of pathological analyses [7]. For malignant tumor that occurs in the aorta, Iwabuchi [8] classified aortic tumors following two types, i.e., intimal or luminal type which main lesion is in the intima, and the mural type which main lesion is in the media to adventitia, since histological classification did not reflect patient prognosis, but tumor locations and shape is clinically important. In particular, the intimal type of tumor when tumor is protruding and exposed to the vessel lumen resulting strong degeneration and necrosis, is diagnosed as intimal sarcoma, because it may be difficult to diagnose pathology correctly due to poor pathological specimen.

Our new classification of tumors of the heart and great vessels are based on the “tumors of the heart and great vessels” AFIP [1] and the WHO classification [4]. The pathological and clinical entities of these tumors were not well established compared with other organs such as tumors of the digestive system, and the WHO classification classified only typical tumor and major cardiac tumors and tumor-like lesions. In our classification, we intended to include almost all the tumors and tumor-like lesions of the heart and great vessels for clinical use based on the idea of the authors. Since this classification is not perfect and did not based on fully pathological aspects, we sincerely hope that the more precise and useful classifications will be established in accordance with the development of studies about tumors of the heart and great vessels.

## Epidemiology and incidence

The cardiovascular tumor is rarely encountered, and its incidence is very low among tumors of all organs. In the past, cardiac tumors are very difficult to diagnose before life and it was often discovered incidentally at autopsy. However, recent advances in diagnostic techniques such as echocardiography and computed tomography (CT) enabled clinical diagnosis during lifetime, and pathological diagnosis of tumor can be obtained by surgically removed tumors or biopsy. From these accumulated pathological and clinical data, it became evident that many varieties of tumors occur in the heart and great vessels as in other organs.

### The incidence of cardiac tumors in autopsy

The incidence of cardiac tumors in autopsy cases has been reported from long time ago, it has been observed difference almost 100 times by the reporter from 0.33 to 0.0017 % so far as in the literatures (Table 4). This wide range of incidence may be due to changes of criteria for pathological diagnosis, transition of subjects for autopsy, and changes of diseases for autopsy [9, 10]. Pollia and Gogol [11] reported the highest incidence of tumors originated from the heart as 0.33 % (154 cases) in autopsy of 46,072 cases, while Straus and Merliss [12] reported the lowest incidence as 0.0017 % (8 cases) in autopsy of 480,000 cases by summarizing the autopsy statistics of six hospitals. Reynen [13] reported the incidence as 0.021 % in 731,309 autopsy cases by summarizing the autopsy statistics from the 22 literatures. It is interesting the presence of year difference of incidence of tumors originated from the heart that incidence of the years 1915–1931 is 0.047 %, while 0.17 % in the years 1954-1979 from the report of Mayo Clinic [10, 14]. In Japan, Mukai et al. [15] from National Cancer Center reported that tumors originated from the heart was found only in 1 case (0.038 %) during the year 1976–1985 among 2,649 autopsy cases who died of cancer. On the other hand, reports of cardiac tumors in children are scarce, Nadas and Ellison [16] reported that incidence of tumors originated from the heart was 0.01 % at autopsy.

**Table 4:** Incidence of primary cardiac tumors diagnosed at autopsy

Authors	Autopsy cases	Primary cardiac tumors	Incidence (%)
Pollia	46,072	154	0.33
Benjamin	40,000	12	0.003
Straus	480,000	8	0.0017
Reynen	731,309	15,357	0.021
Mayo clinic			
Lymburner: 1915–1931	8,550	4	0.047
Wold: 1954–1979	23,673	41	0.17
Mukai	2,649	1	0.038
Nadas			0.01

Modified from reference [10]

### Tumors originated from the heart

The incidence of tumors originated from the heart has changed over the years and the development of medicine between the recent era when the diagnostic methods and surgical resection developed and the era when most of the cardiac tumors were diagnosed by autopsy.

In the AFIP data [3, 17], cardiac myxoma is the most common cardiac tumor accounting for 24–37 % of them, following angiosarcoma (7.3–8.5 %), and papillary fibroelastoma is observed (from 7.9–8.0 %) with approximately the same frequency. Benign tumors are more common in order of myxoma, papillary fibroelastoma, rhabdomyoma, fibroma, hemangioma, and cystic tumor of atrioventricular node, and malignant tumors are more common in order of angiosarcoma, unclassified sarcoma, and malignant fibrous histiocytoma (Table 5). In comparison with adults, the incidence of cardiac tumors in children is very low [3, 16]. In addition, percentages of rhabdomyoma and fibroma are especially high, and histiocytoid cardiomyopathy also is relatively high, but cardiac myxoma which is the most common in adults is characterized as less common [3, 18] (Table 6). Interestingly, rhabdomyoma is considered a specific tumor which in some cases disappears in the course of observation [16]. In children, malignant tumors originated from the heart are very rare, and leiomyosarcoma, unclassified sarcoma, and rhabdomyosarcoma are reported [3, 16].

**Table 5:** Incidence of tumors originated from the heart

Tumors	AFIP—1975	AFIP 1976–1993	Surgical cases
Benign			
Myxoma	130 (24.4 %)	114 (29.5 %)	102 (36.7 %)
Papillary fibroelastoma	42 (7.9 %)	31 (8.0 %)	8 (2.9 %)
Rhabdomyoma	36 (6.8 %)	20 (5.2 %)	6 (2.2 %)
Fibroma	17 (3.2 %)	20 (5.2 %)	18 (6.5 %)
Hemangioma	15 (2.8 %)	17 (4.4 %)	10 (3.6 %)
Lipomatous hypertrophy	0	12 (3.1 %)	7 (2.5 %)
Cystic tumor of AV node	12 (2.3 %)	10 (2.6 %)	0
Granular cell tumor	3 (0.56 %)	4 (1.0 %)	0
Lipoma	45 (8.4 %)	2 (0.5 %)	2 (0.07 %)
Paraganglioma	0	2 (0.5 %)	2 (0.07 %)
Hamartoma	0	2 (0.5 %)	2 (0.07 %)
Histiocytoid cardiomyopathy	0	2 (0.5 %)	0
Inflammatory pseudotumor	0	2 (0.5 %)	2 (0.07 %)
Fibrous histiocytoma	0	1 (0.25 %)	0
Epithelioid hemangioendothelioma	0	1 (0.25 %)	1 (0.04 %)
Pericardial cyst	82 (15.4 %)	0	0
Bronchogenic cyst	7 (1.3 %)	1 (0.25 %)	1 (0.04 %)
Teratoma	14 (2.6 %)	1 (0.25 %)	0
Others	5 (0.94 %)	0	0
Total	408 (76.5 %)	242 (62.7 %)	161 (57.9 %)
Malignant			
Angiosarcoma	39 (7.3 %)	33 (8.5 %)	22 (7.9 %)
Undifferentiated sarcoma	0	33 (8.5 %)	30 (10.8 %)
Malignant fibrous histiocytoma	0	16 (4.1 %)	16 (5.8 %)
Osteosarcoma	5 (0.94 %)	13 (3.4 %)	13 (4.7 %)
Leiomyosarcoma	1 (0.19 %)	12 (3.1 %)	11 (4.0 %)
Fibrosarcoma	14 (2.6 %)	9 (2.3 %)	9 (3.2 %)
Myxosarcoma	0	8 (2.1 %)	8 (2.9 %)
Rhabdomyosarcoma	26 (4.9 %)	6 (1.6 %)	2 (0.07 %)
Synovial sarcoma	1 (0.19 %)	4 (1.0 %)	4 (1.4 %)
Liposarcoma	1 (0.19 %)	2 (0.5 %)	0
Malignant schwannoma	4 (0.75 %)	1 (0.25 %)	1 (0.04 %)
Malignant mesothelioma	19 (3.6 %)	0	0
Others	8 (3.6 %)	0	0
Total	118 (23.5 %)	137 (35.5 %)	116 (41.7 %)
Malignant lymphoma	7 (1.3 %)	7 (1.8 %)	1 (0.04 %)
Total	533	386	278

Modified from references [3, 17]

*AFIP* Armed Forces Institute of Pathology

**Table 6:** Incidence of tumors originated from the heart in infancy (under 16 years)

Tumors	AFIP	<16 years	Becker
	<1 year		<16 years
Benign			
Myxoma	0	4 (7.1 %)	0
Rhabdomyoma	19 (54.3 %)	20 (35.7 %)	9 (42.9 %)
Fibroma	8 (22.9 %)	13 (23.2 %)	5 (23.8 %)
Histiocytoid cardiomyopathy	2 (5.7 %)	2 (3.6 %)	2 (9.5 %)
Hemangioma	1 (2.9 %)	2 (3.6 %)	2 (9.5 %)
Cystic tumor of AV node	1 (2.9 %)	2 (3.6 %)	0
Inflammatory pseudotumor	0	1 (1.8 %)	0
Teratoma	1 (2.9 %)	1 (1.8 %)	0
Lipoma	0	0	1 (4.8 %)
Total	32 (91.3 %)	45 (80.4 %)	19 (90.5 %)
Malignant			
Rhabdomyosarcoma	1 (2.9 %)	3 (5.4 %)	0
Angiosarcoma	0	1 (1.8 %)	0
Undifferentiated sarcoma	1 (2.9 %)	3 (5.4 %)	1 (4.8 %)
Malignant fibrous histiocytoma	1 (1.8 %)	0	
Leiomyosarcoma	1 (2.9 %)	1 (1.8 %)	1 (4.8 %)
Fibrosarcoma	0	1 (1.8 %)	0
Myxosarcoma	0	1 (1.8 %)	0
Total	3 (8.7 %)	11 (19.6 %)	2 (9.5 %)
Total	35	56	21

Modified from references [3, 18]

*AFIP* Armed Forces Institute of Pathology

In this way, the susceptible age when cardiac tumors have been diagnosed is different for each tumor. According to the AFIP data [3], rhabdomyoma and teratoma occur in infancy, rhabdomyosarcoma and cardiac fibroma is common in the adolescence, mesothelioma, lipomatous hyperplasia, and papillary fibroelastoma tended to occur in the elderly [3, 9, 19] (Table 7). Our results of research about cardiac tumors in Japan supported by the Grant-in-aid for Scientific Research from Japan Society for the Promotion of Science (JSPS: 21591793) is not similar to that of the data of AFIP for susceptible age when cardiac tumors were diagnosed. This may be due to the small number of cases in our study compared to AFIP data and recent high incidences of presentation of cardiac tumors in the elderly in recent aging society.

**Table 7:** Susceptible age when cardiac tumors have been diagnosed

Tumors	AFIP	Endo (years)	Amano (years)
Teratoma	16 weeks		
Rhabdomyoma	33 weeks		
Fibroma	13 years		
Rhabdomyosarcoma	15 years		50.5
Hemangioma	31 years		
Cystic tumor of atrioventricular node	33 years		
All sarcomas	41 years		67.2
Myxoma	50 years	58.5	64.0
Mesothelioma	57 years		
Angiosarcoma			57.4
Papillary fibroelastoma	59 years		70.9
Lipomatous hypertrophy	64 years		
Leiomyosarcoma			69.8

Modified from reference [9]

*AFIP* Armed Forces Institute of Pathology

Cardiac tumors have been reported frequently in the regional meetings of the Japanese Association for Thoracic Surgery (JATS) and the Japanese Circulation Society (JCS). Compiling the tumor incidence by reported cases that have been reported in the meeting of regional meeting of JATS and JCS during the years 1999–2010, the most frequent benign tumor was cardiac myxoma (34.0–43.1 %), followed by papillary fibroelastoma (11.4–17.7 %), lipoma (2.4–3.4 %), and hemangioma (2.4–2.6 %) in the order [9] (Table 8). On the other hand, frequency of the malignant cardiac tumor with the exception of malignant lymphoma was angiosarcoma (8.2–9.5 %), malignant fibrous histiocytoma (3.3–4.3 %), and leiomyosarcoma (1.9–2.2 %) in the order. Although the approximate frequency rate of cardiac tumors were similar among regional meeting of JATS and JCS, it may be possible that the same case have been reported in both meetings. However, for malignant lymphoma, different incidence between JCS: 20.4 % and JATS: 6.0 % may reflect that radiation therapy and chemotherapy is the subject of treatment and surgery have been attempted to patients only for open biopsy due to failure of non-invasive tissue diagnosis or for life-saving surgery for patients suffering from heart failure due to intracavitary obstructing tumor. In addition, since the relatively rare cases tend to be reported at such regional meetings, it is plausible that these data did not reflect the actual incidence of tumor in Japan.

**Table 8:** Reported incidences in the regional meeting of JATS and JCS (1999–2010)

Tumors	JCS	JATS
Benign		
Myxoma	125 (34.0 %)	100 (43.1 %)
Papillary fibroelastoma	42 (11.4 %)	41 (17.7 %)
Lipoma	9 (2.4 %)	8 (3.4 %)
Hemangioma	9 (2.4 %)	6 (2.6 %)
Fibroma	5 (1.4 %)	5 (2.2 %)
Lipomatous hypertrophy	3 (0.8 %)	2 (0.9 %)
Rhabdomyoma	1 (0.3 %)	0
Paraganglioma	1 (0.3 %)	2 (0.9 %)
Neurinoma	1 (0.3 %)	1 (0.45 %)
Hamartoma	1 (0.3 %)	0
Lipomatous hamartoma of cardiac values	1 (0.3 %)	0
Inflammatory pseudotumor	0	1 (0.45 %)
Angiomyolipoma	0	1 (0.45 %)
Total	198 (53.8 %)	167 (72.0 %)
Malignant		
Angiosarcoma	30 (8.2 %)	22 (9.5 %)
Malignant fibrous histiocytoma	12 (3.3 %)	10 (4.3 %)
Leiomyosarcoma	7 (1.9 %)	5 (2.2 %)
Liposarcoma	4 (1.1 %)	2 (0.9 %)
Osteosarcoma	3 (0.8 %)	3 (1.3 %)
Synovial sarcoma	3 (0.8 %)	2 (0.9 %)
Rhabdomyosarcoma	3 (0.8 %)	1 (0.45 %)
Fibrosarcoma	2 (0.3 %)	0
Chondrosarcoma	0	1 (0.45 %)
Malignant schwannoma	0	1 (0.45 %)
Endothelial sarcoma	0	2 (0.9 %)
Epithelioid hemangioendothelioma	0	1 (0.45 %)
Others	31 (8.4 %)	1 (0.45 %)
Total	95 (25.8 %)	51 (22.0 %)
Malignant lymphoma	75 (20.4 %)	14 (6.0 %)
Total	368	232

Modified from reference [9]

*JCS* Japanese Circulation Society, *JATS* Japanese Association for Thoracic Surgery

**Fig. 1:**
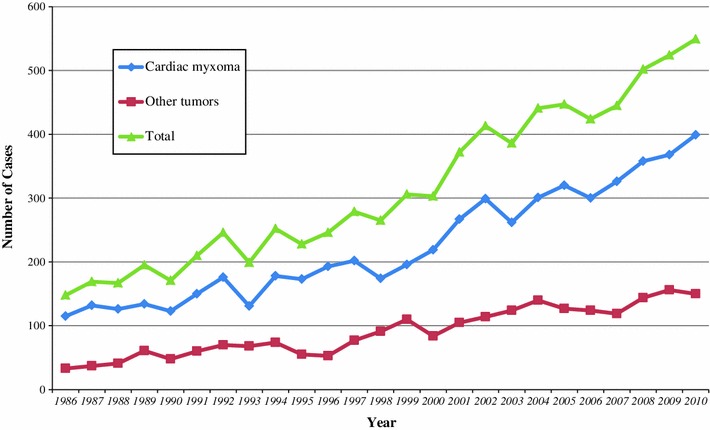
Changes of cases of reported cardiac tumors by the annual report by The Japanese Association for Thoracic Surgery

Regarding number of cases of surgery for cardiac tumors in Japan, there are annual statistics of JATS; since the survey began in 1986, incidence of cardiac tumor has been increasing every year. Recently, about 400–500 cases have been operated in every year, and cardiac myxoma accounts for about 70 % of them (Fig. 1).

By our research project for cardiac tumors in Japan supported by the Grant-in-aid for scientific research from Japan Society for the Promotion of Science, we send out questionnaires to the JATS- and JCS-certified 1,789 hospitals in 2010 asking the cardiac tumor incidence between January and December, 2009. As a result, 580 cases with cardiovascular tumors have been experienced in 326 hospitals, most of 389 cases (67.1 %) were benign tumors originated from the heart, following malignant tumors originated from the heart were 90 cases (15.5 %) and metastatic tumors were 75 cases (12.9 %) [9] (Table 9). In addition, 12 cases of pericardial tumor (2.1 %), and 14 cases of tumor of the great vessels (2.4 %) have been experienced. Considering the annual statistics from JATS and this clinical epidemiological study in Japan, it is estimated that the tumors originated from the heart occurs near 500 cases per year. In this study, tumors originated from the heart have been reported 489 cases. Among these cases, the 390 cases (79.8 %) of benign cardiac tumors were more than malignancy, and 332 cases (67.8 %) were cardiac myxoma, followed by 46 cases of papillary fibroelastoma (9.4 %), 5 cases of rhabdomyoma (1.0 %), lipomatous hyperplasia, lipoma, granular cell tumor, paraganglioma, schwannoma, hamartoma, and perivascular epithelioid cell tumor. Eighty-eight cases (18.0 %) were malignant tumors originated from the heart, among them 43 cases (8.9 %) were malignant lymphoma, followed 9 cases (3.5 %) of angiosarcoma, 17 cases (1.8 %) of rhabdomyosarcoma, 5 cases (1.0 %) of malignant fibrous histiocytoma, and 2 cases (0.4 %) of osteosarcoma. As of pericardial tumor, two cases of malignant mesothelioma were reported. Tumors of the great vessels were extremely rare. Pulmonary artery tumors were most frequent, among them intimal sarcoma in five cases, angiosarcoma in two cases, and one case of leiomyosarcoma. Two cases of metastatic aortic tumors (thymic carcinoma, esophageal carcinoma) were reported, and in the vena cava tumors, one case of primary angiosarcoma and three metastatic tumors were reported.

**Table 9:** Incidence of tumors originated from the heart and the pericardium in Japan (2009)

Tumors	Cases
Benign	
Myxoma	332 (69.9 %)
Papillary fibroelastoma	46 (9.7 %)
Rhabdomyoma	5 (1.1 %)
Lipomatous hypertrophy	1 (0.2 %)
Lipoma	1 (0.2 %)
Granular cell tumor	1 (0.2 %)
Paraganglioma	1 (0.2 %)
Neurinoma	1 (0.2 %)
Hamartoma	1 (0.2 %)
PEComa	1 (0.2 %)
Total	390 (82.1 %)
Malignant	
Angiosarcoma	14 (3.0 %)
Malignant fibrous histiocytoma	5 (1.1 %)
Rhabdomyosarcoma	4 (0.8 %)
Leiomyosarcoma	4 (0.8 %)
Osteosarcoma	2 (0.4 %)
Synovial sarcoma	1 (0.2 %)
Total	30 (6.3 %)
Malignant lymphoma	43 (9.1 %)
Unknown	10 (2.1 %)
Pericardial tumor	
Malignant mesothelioma	2 (0.4 %)
Total	475

Modified from reference [9]

*PEComa* perivascular epithelioid cell tumor

### Metastatic cardiac tumors

Pathway of metastasis from other organs to the heart or pericardium are direct invasion from nearby organs such as lung, mediastinum or esophagus, distant metastasis by lymphatic metastasis or hematogenous metastasis, extension of the tumor via vena cava or pulmonary vein to the heart chamber, or combination of these routes.

The incidence of metastatic cardiac tumors in autopsy cases has been reported 0.7–3.5 % and in autopsy cases of malignant tumor has been reported 1.7–14 %; thus metastatic cardiac tumors are more frequent than malignant tumors originated from the heart [20] (Table 10). Lymburner [14] reported high incidence of metastatic cardiac tumors as 52 cases (0.61 %) and malignant tumors originated from the heart as 4 cases (0.047 %) in 8,550 autopsy cases, which ratio is 13:1 in 1934.

**Table 10:** Reported incidences of metastatic cardiac tumors

Authors	Years	Autopsy cases	Cancer cases	Cardiac metastasis	Incidence (%)	
					In cancer cases	In autopsy cases
1917–1951						
Symmers	1917	298		5	1.7	
Willis	1933	323		20	6.2	
Burke	1934	327		14	4.3	
Pollia	1936	1,450		29	2.0	
Scott	1939	1,082		101	9.3	
Ritchie	1941	857		16	1.9	
Prichard	1951	4,375		146	3.3	
Total		8,712		331	3.8	
1975–2007						
Abioye	1975	6,064	752	64	8.5	1.1
Ambrosio	1980	2,222	590	38	6.4	1.7
Xiong	1986	2,050	484	68	14.0	3.3
Karwinski	1989	8,571	2,833	130	4.6	1.5
MacGee	1991	2,455	1,311	57	4.4	2.4
Silvestri	1997	4,769	1,928	162	8.4	3.4
Abraham	1999	3,314	806	95	11.8	2.9
Rafajlovski	2005	11,403	2,928	79	2.7	0.7
Bussani	2007	18,751	7,289	662	9.1	3.5
Total		59,599	18,921	1,355	7.1	2.3

Modified from reference [20]

Mukai et al. [15] reported that during a 10-year period (1976–1985), only one case of a primary malignant mesothelioma (0.038 %) was identified among 2,649 autopsies of malignant tumors at the National Cancer Center Hospital, in contrast, there were 407 cases (15.4 %) in which heart and/or pericardium were secondarily involved with a malignant tumor from other organs. Burke indicated that if one accepts a rate of 1 to 3 % for cardiac metastases occur in autopsy, and a rate of 1 to 3 % for malignant tumors originated from the heart seen in autopsy, then the ratio of cardiac metastases to malignant tumors originated from the heart is 100:1 to 1,000:1. They suggested that this estimate is so higher than previous estimate 1:20 to 1:40; thus, a ratio that falls within the range of 100 to 1,000:1 is likely an accurate estimate of the true ratio of metastatic tumors to malignant tumors originated from the heart [3]. Since Butany et al. [21] reported that two malignant tumors originated from the heart (0.017 %) and 264 metastatic tumors (2.31 %) were identified among 11,432 autopsies (the ratio of malignant tumors originated from the heart to metastatic tumors 1:132), Burke’s estimate is plausible.

Lung cancer is the most high incident metastatic cardiac tumor followed by breast cancer, malignant melanoma, leukemia, and malignant lymphoma [15, 21] (Table 11). In our study about cardiac tumors in Japan supported by the Grant-in-aid for scientific research revealed that among 116 metastatic tumors, direct invasion to the heart was 32 cases (27.6 %), distant metastasis from other organ cancer was 39 cases (34.5 %), and intravenous extension of the tumor was 33 cases (28.4 %) (Table 12). As the rate of metastases to the heart and pericardium (number of cases with metastases to the heart/number of autopsy of malignancy for each ×100), malignant melanoma most frequently metastasizes to the heart (49–67.5 %), followed by germ cell tumors (43 %), leukemia (21.4–34 %), malignant lymphoma (18.4–21 %), and others [3, 15] (Table 13). Bussani et al. [22] reported the study for an autopsy of 18,751 cases, and among 7,289 cases of malignant tumor metastasis to the heart was found in 622 cases (9.1 %). Malignant pleural mesothelioma showed the highest rate (48.4 %) of metastasis, followed by malignant melanoma (27.8 %) and lung adenocarcinoma (21 %). In addition, the frequency rate of lung cancer was different in accordance with the pathology of the lung cancer, and in adenocarcinoma it was 21 %, undifferentiated carcinoma 19.5 %, squamous cell carcinoma 18.2 %, and bronchoalveolar carcinoma 9.8 %.

**Table 11:** Metastatic cardiac and pericardial tumors

Primary lesions		Mukai		Butany
	Heart	pericardium	Total (%)	(%)
Lung	104	32	136 (33.4)	89 (33.7)
Breast	27	8	35 (8.6)	31 (11.7)
Stomach	22	8	30 (7.4)	3 (1.1)
Malignant lymphoma	25	2	27 (6.6)	24 (9.1)
Esophagus	18	7	25 (6.1)	8 (3.0)
Leukemia/Myeloma	20	2	22 (5.4)	32 (12.1)
Uterus	18	2	20 (4.9)	
Malignant melanoma	19		19 (4.7)	8 (3.0)
Sarcoma	14	3	17 (4.2)	
Rectum	9	3	12 (2.9)	9 (3.4)
Tongue	9	2	11 (2.7)	
Germ cell tumor	8	1	9 (2.2)	
Thyroid	6		6 (1.5)	1 (0.4)
Kidney	4	1	5 (1.2)	3 (1.1)
Oral cavity	4		4 (1.0)	3 (1.1)
Salivary gland	3	1	4 (1.0)	
Larynx	1	2	3 (1.0)	1 (0.4)
Pharynx	1	2	3 (1.0)	
Thymus	3		3 (1.0)	
Skin	2		2 (0.5)	
Bile duct	2		2 (0.5)	
Pancreas	2		2 (0.5)	10 (3.8)
Urinary bladder	2		2 (0.5)	
Unknown	2		2 (0.5)	16 (6.1)
Neuroblastoma	1		1 (0.5)	
Nasal/paranasal sinus	1		1 (0.25)	
Small intestine				1 (0.4)
Liver	1		1 (0.25)	2 (0.8)
Prostate	1		1 (0.25)	
Ovarium	1		1 (0.25)	
External genitalia	1		1 (0.25)	
Malignant melanoma				5 (1.9)
Reproductive organ				6 (2.3)
Soft tissue				8 (3.0)
Mediastinum				2 (0.8)
Brain				2 (0.8)
Total	324	83	407	264

Modified from reference [9]

**Table 12:** Incidence of metastasis to the heart and the pericardium in Japan (2009)

Tumors	Cases
Heart	
Direct invasion	32 (27.6 %)
Mediastinal tumors	11 (9.5 %)
Thymic cancer	8 (6.9 %)
Germ cell tumor	1
Malignant lymphoma	1
Unknown	1
Lung cancer	19 (16.4 %)
Esophageal cancer	2 (1.7 %)
Distant metastasis	39 (34.5 %)
Thyroid cancer	5 (4.3 %)
Breast cancer	3 (2.6 %)
Lung cancer	4 (3.4 %)
Gastrointestinal cancer	12 (10.3 %)
Esophageal cancer	2 (1.7 %)
Gastric cancer	3 (2.6 %)
Colon cancer	1
Hepatoma	3 (2.6 %)
Unknown	3 (2.6 %)
Kidney cancer	4 (3.4 %)
Seminoma	1
Germ cell tumor	1
Urinary bladder cancer	1
Uterus cancer	1
Osteosarcoma	1
Soft tissue tumor	1
Unknown	5 (4.3 %)
Intravascular extension	33 (28.4 %)
Renal cancer	24 (20.7 %)
Uterine tumor	3 (2.6 %)
Colon cancer	3 (2.6 %)
Hepatoma	2 (1.7 %)
Thyroid cancer	1
Ureteral cancer	1
Pericardium	12 (10.3 %)
Malignant thymoma	3 (2.6 %)
Leukemia	3 (2.6 %)
Malignant lymphoma	2 (1.7 %)
Breast cancer	1
Malignant mesothelioma	1
Lung cancer	1
Esophageal cancer	1
Total	116

Modified from references [9]

**Table 13:** Frequency of metastasis to the heart and the pericardium in autopsy cases

		AFIP					Mukai	
Tumors	Autopsy	heart	Pericardium	Total (%)	Autopsy	Heart	Pericardium	Total (%)
Malignant melanoma	69	32	2	34 (49)	28	19	0	19 (67.5)
Germ cell tumor	21	8	1	9 (43)	21	8	1	9 (42.9)
Leukemia	202	66	2	68 (34)	103	20	2	22 (21.4)
Lung cancer	1,037	180	112	292 (28)	484	04	32	136 (28.1)
Sarcoma	159	24	11	35 (22)	90	14	3	17 (18.9)
Malignant lymphoma	392	67	15	82 (21)	147	25	2	27 (18.4)
Breast cancer	685	70	69	139 (20)	193	27	8	35 (18.1)
Esophageal cancer	294	37	13	50 (17)	187	18	7	25 (13.4)
Renal cancer	114	12	5	17 (15)	61	4	1	5 (8.2)
Oral cavity/tongue cancer	235	22	2	24 (10)	104	16	3	19 (18.3)
Laryngeal cancer	100	9	2	11 (11)	67	1	2	3 (4.5)
Thyroid cancer	97	9	3	12 (12)	77	6	0	6 (7.8)
Uterine cancer	451	36	5	41 (9)	195	18	2	20 (10.3)
Gastric cancer	603	28	16	44 (7)	391	22	8	30 (7.7)
Colon cancer	440	22	3	25 (6)	167	9	3	12 (7.2)
Pharyngeal cancer	67	1	2	3 (4.5)	34	1	2	3 (8.8)
Urinary bladder cancer	128	8	0	8 (6)	47	2	0	2 (4.3)
Ovarian cancer	188	2	6	8 (4)	49	1	0	1 (2.0)
Prostate cancer	171	6	0	6 (4)	48	1	0	1 (2.0)
Nasal/paranasal sinus cancer	32	1	0	1 (3)	32	1	0	1 (3.1)
Pancreatic cancer	185	6	0	6 (3)	89	2	0	2 (2.2)
Hepato-biliary cancer	325	7	0	7 (2)	250	3	2	5 (2.0)
Malignant thymoma					5	3	0	3 (60.0)
Skin cancer					14	2	0	2 (14.3)
External genital cancer					7	0	1	1 (14.3)
Neuroblastoma					11	0	1	1 (9.1)
Unknown					9	2	0	2 (22.2)
Others					82	0	0	0
Total	6,240	654	299	953 (15)	2,992	324	83	407 (13.6)

Modified from reference [9]

*AFIP* Armed Forces Institute of Pathology

The metastatic site of the metastatic tumor in the heart are to the heart, pericardium, and both heart and pericardium. Mukai et al. [15] reported that among 407 cases of cardiac metastasis, metastasis to the myocardium was 169 cases (41.5 %), followed by epicardium 136 cases (33.4 %), pericardium 78 cases (19.2 %), and endocardium 24 cases (5.9 %), and Butany et al. [21] reported that among 193 cases of cardiac metastasis, metastasis to the pericardium was 127 cases (65.8 %), myocardium 56 cases (29.0 %), epicardium 48 cases (24.9 %), and endocardium 6 cases (3.1 %) [12, 21] (Table 14). The differences of the incidence of metastatic cardiac tumor by reporters may be due to what kind of hospital, i.e., general hospital, cancer specialized hospital or cardiovascular specialized hospital, or whether hospital has system to perform an autopsy and department pathology or not [21]. It is also possible that the statistical results are different depending on whether to include the hematopoietic tumors such as leukemia and malignant lymphoma. While there is a report that incidence of metastatic cardiac tumor does not significantly change with time [23], Mukai et al. [15] reported increase in its incidence from 9.7 % in 1996 report to 15.4 % in 1988. In addition, according to the study of the reported cases summarized by Al-Mamgani et al. [20], its incidence prior to 1975 was 3.8 % and subsequent significant increased to 7.1 % in recent years.

**Table 14:** Metastatic sites of the heart and the pericardium

Sites	Mukai	Butany
Heart/pericardium	407	
Heart: with or without pericardial involvement	329	
Pericardium only	78	
Extent of involvement		
Pericardium	78 (19.2 %)	127 (65.8 %)
Epicardium	136 (33.4 %)	48 (24.9 %)
Myocardium	169 (41.5 %)	56 (29.0 %)
Endocardium	4 (5.9 %)	6 (3.1 %)
Localization of involvement		
Right side only	34	
Left side only	50	
Septum only	10	
Bilateral or diffuse	52	
Intracavitary tumor thrombus	7	

Modified from reference [9]

Formerly, most of the metastatic cardiac tumors have been so far reported in autopsy cases. Recent advances of diagnostic imaging methods by CT, MRI, and echocardiography enabled to discover metastasis to the heart early even in the absence of symptoms related to the heart, such as arrhythmia and heart failure [24]. In addition to these development of diagnostic tools, incidence of metastatic cardiac tumors may increase further by life-prolonging effect by anti-cancer drugs .
