# Erratum to: Clinical classification of cardiovascular tumors and tumor-like lesions, and its incidences

**DOI:** 10.1007/s11748-013-0244-2

**Published:** 2013-05-21

**Authors:** Jun Amano, Jun Nakayama, Yasuo Yoshimura, Uichi Ikeda

**Affiliations:** 1 Department of Cardiovascular Surgery, Shinshu University School of Medicine, 3-1-1 Asahi, Matsumoto, Nagano Japan; 2 Department of Molecular Pathology, Shinshu University Graduate School of Medicine, 3-1-1 Asahi, Matsumoto, Nagano Japan; 3 Department of Orthopaedic Surgery, Shinshu University School of Medicine, 3-1-1 Asahi, Matsumoto, Nagano Japan; 4 Department of Cardiovascular Medicine, Shinshu University School of Medicine, 3-1-1 Asahi, Matsumoto, Nagano Japan

## Erratum to: Gen Thorac Cardiovasc Surg DOI 10.1007/s11748-013-0214-8

Errors appeared in the above-cited article. In the Author Biography on the last page of the article, the author’s affiliation should read “Shinshu University School of Medicine”, not “Shinshu University Faculty of Medicine”.

In addition, in Table 6, the column headings should appear as follows.

**Table 6:** Incidence of tumors originated from the heart in infancy (under 16 years)

Tumors	AFIP		Becker
	<1 year	<6 years	<16 years
Benign			
Myxoma	0	4 (7.1 %)	0
Rhabdomyoma	19 (54.3 %)	20 (35.7 %)	9 (42.9 %)
Fibroma	8 (22.9 %)	13 (23.2 %)	5 (23.8 %)
Histiocytoid cardiomyopathy	2 (5.7 %)	2 (3.6 %)	2 (9.5 %)
Hemangioma	1 (2.9 %)	2 (3.6 %)	2 (9.5 %)
Cystic tumor of AV node	1 (2.9 %)	2 (3.6 %)	0
Inflammatory pseudotumor	0	1 (1.8 %)	0
Teratoma	1 (2.9 %)	1 (1.8 %)	0
Lipoma	0	0	1 (4.8 %)
Total	32 (91.3 %)	45 (80.4 %)	19 (90.5 %)
Malignant			
Rhabdomyosarcoma	1 (2.9 %)	3 (5.4 %)	0
Angiosarcoma	0	1 (1.8 %)	0
Undifferentiated sarcoma	1 (2.9 %)	3 (5.4 %)	1 (4.8 %)
Malignant fibrous histiocytoma	1 (1.8 %)	0	
Leiomyosarcoma	1 (2.9 %)	1 (1.8 %)	1 (4.8 %)
Fibrosarcoma	0	1 (1.8 %)	0
Myxosarcoma	0	1 (1.8 %)	0
Total	3 (8.7 %)	11 (19.6 %)	2 (9.5 %)
Total	35	56	21

Modified from references [3, 18]

*AFIP* Armed Forces Institute of Pathology

